# Supplementary material for: Similar image search for histopathology: SMILY
Source: NPJ Digit Med. 2019 Jun 21;2:56. doi: 10.1038/s41746-019-0131-z (PMC6588631; doi:10.1038/s41746-019-0131-z)
Supplement: Supplementary file 1 — Supplementary Materials [file 41746_2019_131_MOESM1_ESM.pdf]

Supplementary Figures

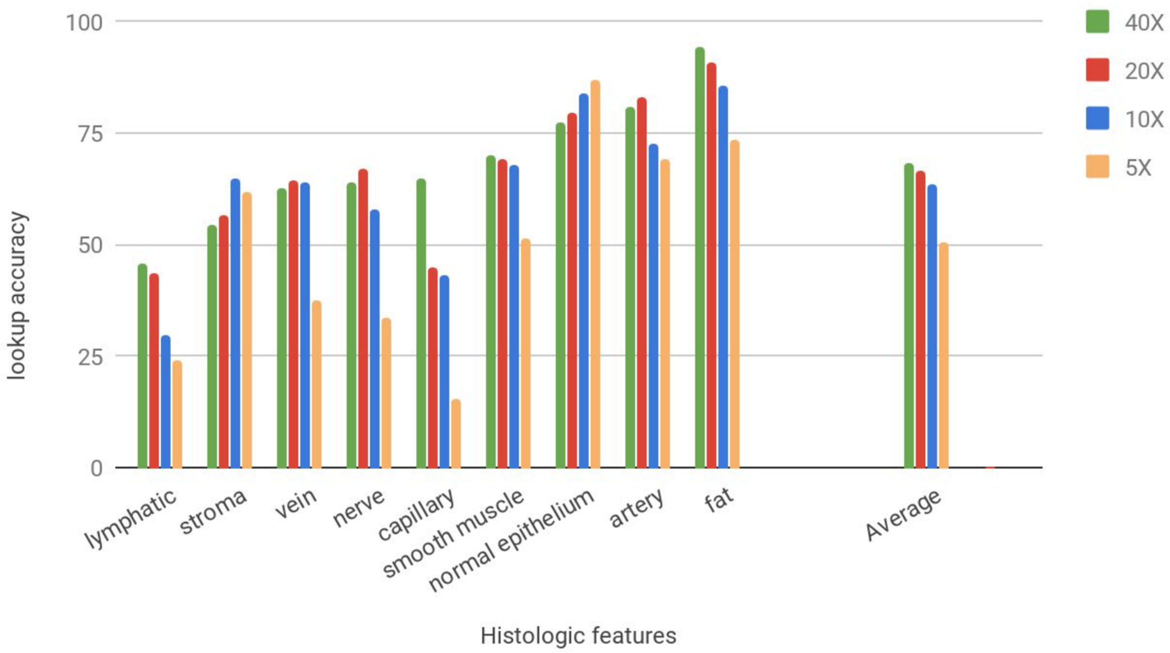

Supplementary Fig. 1 | Top-5 score for histology match using SMILY at different magnifications. The 5X magnification results are lower because there are substantially fewer image patches per slides at that magnification, of which even fewer pass the 1,000-pixel filter used to enhance search result diversity.

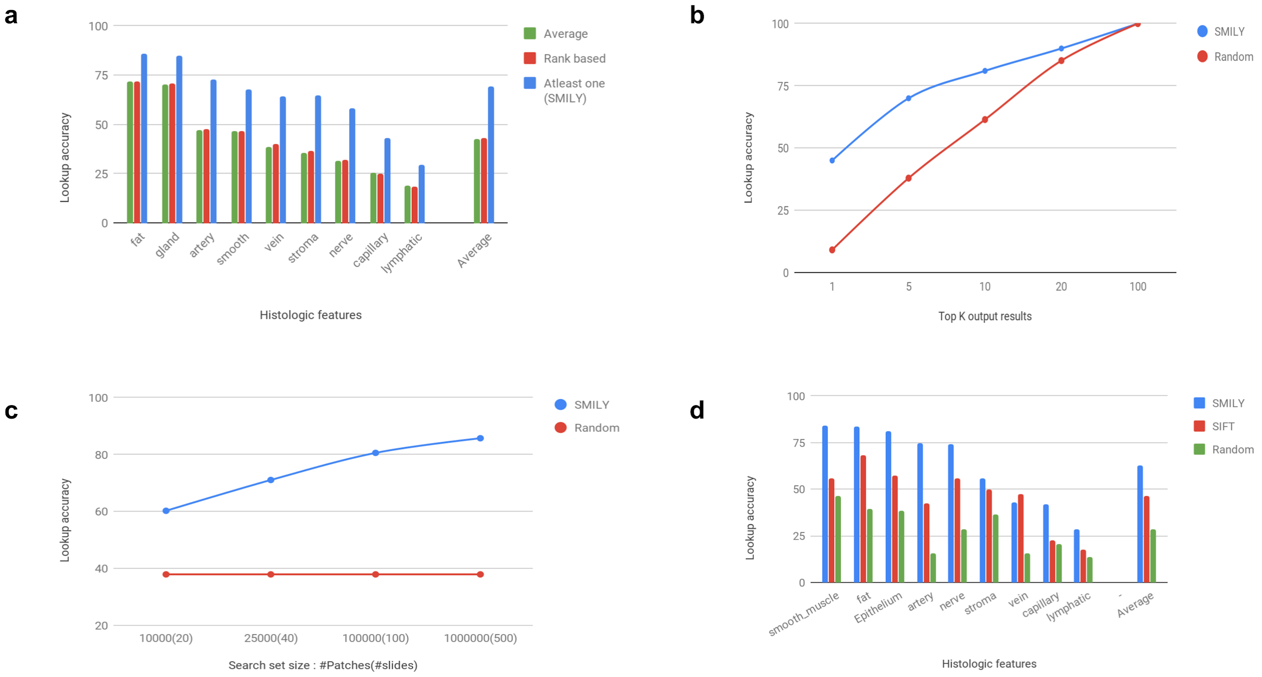

Supplementary Fig. 2 | Additional performance metrics for SMILY. (a) Comparison different metrics: averaging the histology match (1/0), weighted based on rank in the search results, and at-least-one (top-5 score). **(b)** Comparison of different values of “k” in the top-k score (results for histologic feature match in prostate specimens). **(c)** Effect of search database size (results for gleason cancer grade match in prostate specimens). **(d)** Top-5 score when searching the original annotations in prostate specimens (without resample to ensure uniform distribution of histologic features).

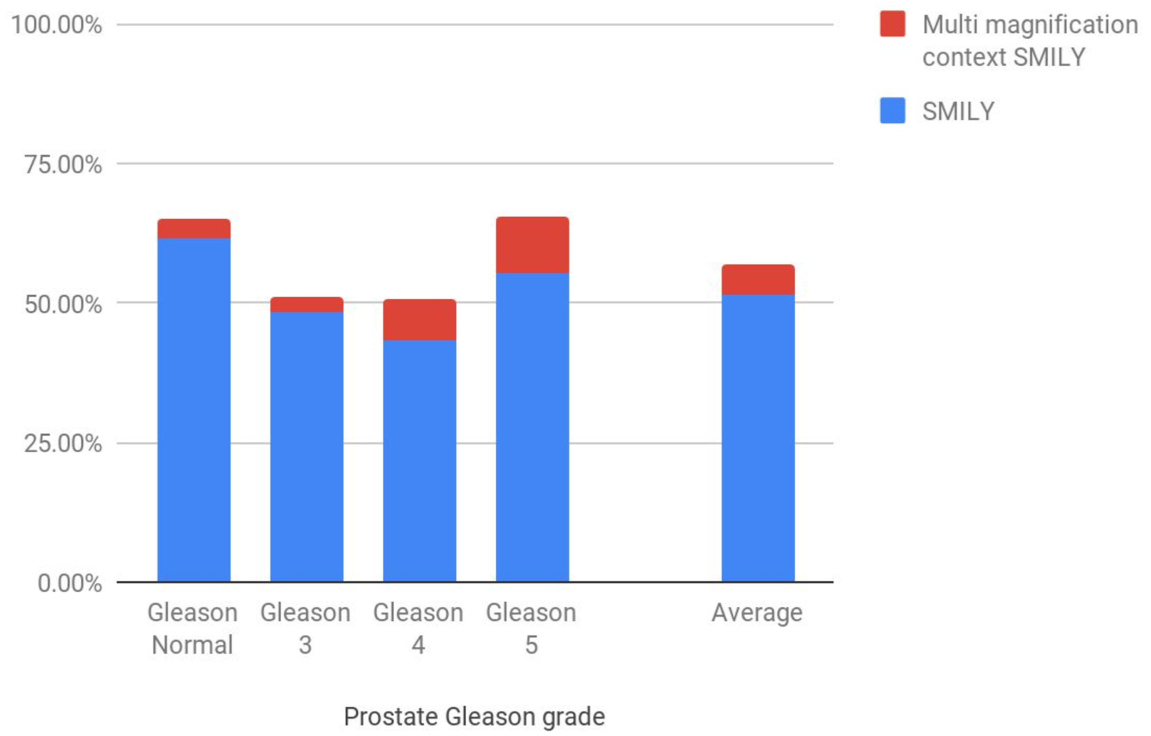

Supplementary Fig. 3 | Comparing the use of a single magnification (in blue) versus multiple magnifications simultaneously (in red) for retrieving patches with similar Gleason patterns. For a query patch at 10X magnification, the multi-magnification embedding is the concatenation of embeddings computed at 5X, 10X, and 4 image patches at 20X (to capture the entire 10X image). For better accuracy, these experiments were run using a larger dataset[3] (that did not have histologic feature annotations) than other results presented in this paper.

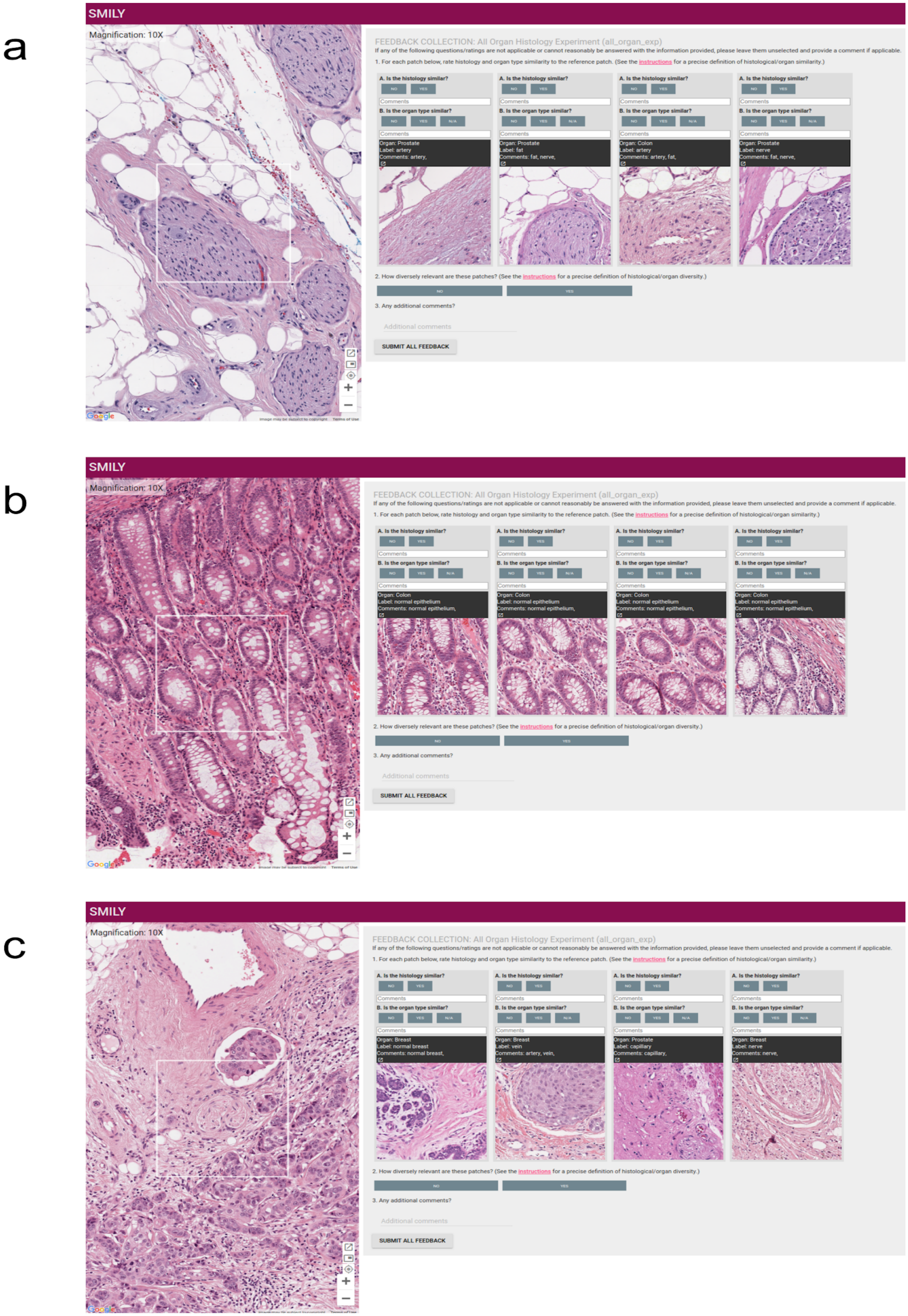
a
SMILY
Magnification: 10X
FEEDBACK COLLECTION: All Organ Histology Experiment (all_organ_exp)
If any of the following questions/ratings are not applicable or cannot reasonably be answered with the information provided, please leave them unselected and provide a comment if applicable.
1. For each patch below, rate histology and organ type similarity to the reference patch. (See the instructions for a precise definition of histological/organ similarity.)
A. Is the histology similar?
NO
YES
Comments
B. Is the organ type similar?
NO
YES
N/A
Comments
Organ: Prostate
Label: artery
Comments: artery,
Organ: Prostate
Label: fat
Comments: fat, nerve,
Organ: Colon
Label: artery
Comments: artery, fat,
Organ: Prostate
Label: nerve
Comments: fat, nerve,
2. How diversely relevant are these patches? (See the instructions for a precise definition of histological/organ diversity.)
NO
YES
3. Any additional comments?
Additional comments
SUBMIT ALL FEEDBACK
b
SMILY
Magnification: 10X
FEEDBACK COLLECTION: All Organ Histology Experiment (all_organ_exp)
If any of the following questions/ratings are not applicable or cannot reasonably be answered with the information provided, please leave them unselected and provide a comment if applicable.
1. For each patch below, rate histology and organ type similarity to the reference patch. (See the instructions for a precise definition of histological/organ similarity.)
A. Is the histology similar?
B. Is the organ type similar?
Organ: Colon
Label: normal epithelium
Comments: normal epithelium,
2. How diversely relevant are these patches? (See the instructions for a precise definition of histological/organ diversity.)
3. Any additional comments?
Additional comments
SUBMIT ALL FEEDBACK
c
SMILY
Magnification: 10X
FEEDBACK COLLECTION: All Organ Histology Experiment (all_organ_exp)
If any of the following questions/ratings are not applicable or cannot reasonably be answered with the information provided, please leave them unselected and provide a comment if applicable.
1. For each patch below, rate histology and organ type similarity to the reference patch. (See the instructions for a precise definition of histological/organ similarity.)
A. Is the histology similar?
B. Is the organ type similar?
Organ: Breast
Label: normal breast
Comments: normal breast,
Organ: Breast
Label: vein
Comments: artery, vein,
Organ: Prostate
Label: capillary
Comments: capillary,
Organ: Breast
Label: nerve
Comments: nerve,
2. How diversely relevant are these patches? (See the instructions for a precise definition of histological/organ diversity.)
3. Any additional comments?
Additional comments
SUBMIT ALL FEEDBACK

Supplementary Fig. 4 | Samples of SMILY similar image retrieval (a) Query consists of a nerve from a prostate specimen, alongside stromal connective tissue, adipocytes, and blood vessels. The search results contained (1) connective tissue adjacent to a blood vessel, (2) a nerve adjacent to fat, (3) a blood vessel adjacent to fat, and (3) a nerve adjacent to fat and connective tissue. **(b)** Query consists of nerve, stroma, tumor, and a lymphatic vessel containing metastatic tumor from a breast specimen. The search results contained (1) a large tumor focus and stroma, (2) a nerve adjacent to stroma and muscle connective tissue, (3) a lymphatic vessel with stroma, and (4) a nerve adjacent to stroma and a blood vessel. **(c)** Query consists of colonic glands cut in cross and longitudinal sections. The search results all contained colonic glands with similar architecture.

a

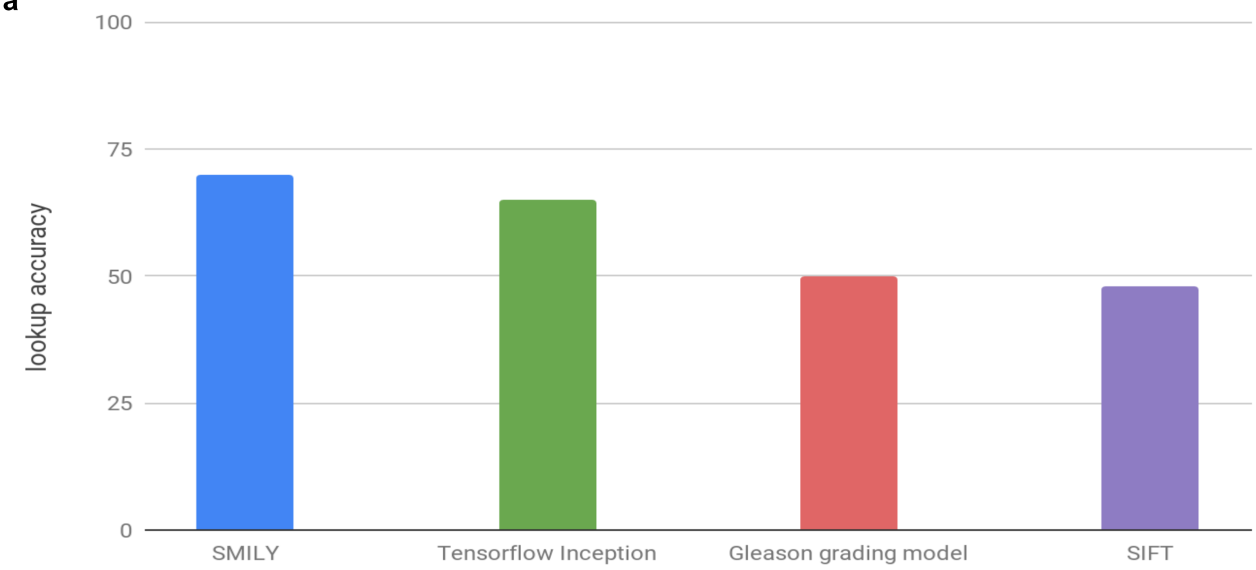

b

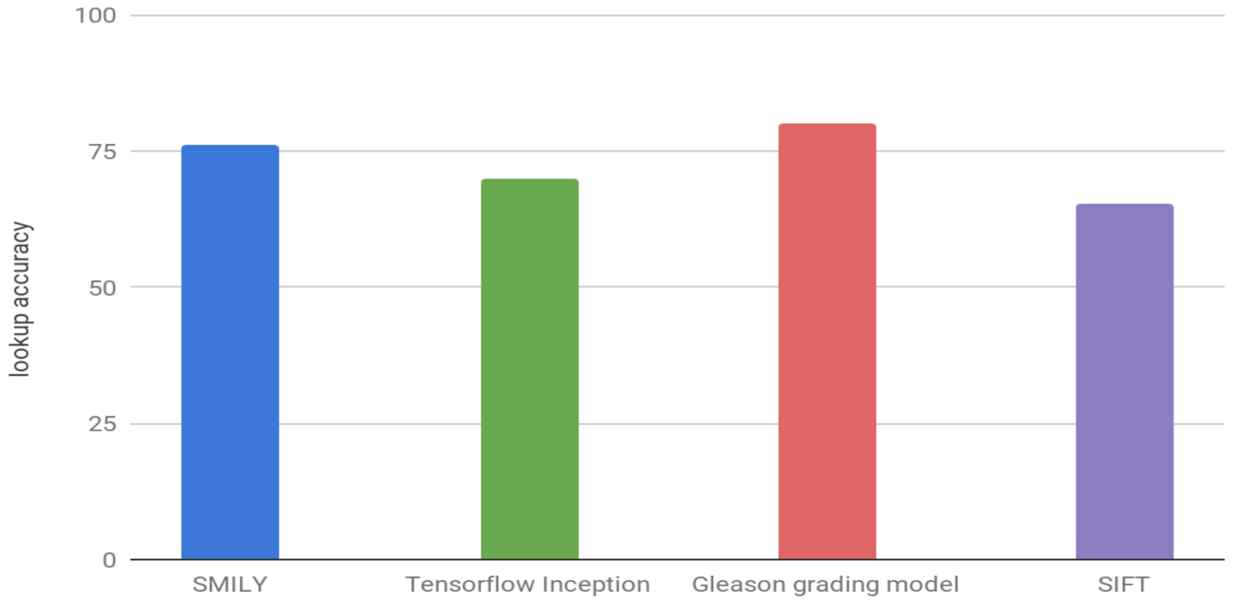

Supplementary Fig. 5 | Comparison of using different pre-trained embedding generators: SMILY's deep ranking network, Inception (V3)[1,2], a network trained for Gleason grading[3], and scale-invariant feature transform (SIFT)[4] for retrieving **(a)** image patches with similar histologic features and **(b)** image patches with similar Gleason pattern.

Supplementary References

1. Szegedy, C., Vanhoucke, V., Ioffe, S., Shlens, J. & Wojna, Z. Rethinking the Inception Architecture for Computer Vision. *arXiv [cs.CV]* (2015).
2. TensorFlow Hub. Available at: https://tfhub.dev/google/imagenet/inception_v3/classification/1. (Accessed: 23rd January 2019)
3. Nagpal, K. *et al.* Development and Validation of a Deep Learning Algorithm for Improving Gleason Scoring of Prostate Cancer. *arXiv [cs.CV]* (2018).
4. Mehta, N., Alomari, R. S. & Chaudhary, V. Content based sub-image retrieval system for high resolution pathology images using salient interest points. *Conf. Proc. IEEE Eng. Med. Biol. Soc.* **2009**, 3719–3722 (2009).
