# Supplementary material for: Similar image search for histopathology: SMILY
Source: NPJ Digit Med. 2019 Jun 21;2:56. doi: 10.1038/s41746-019-0131-z (PMC6588631; doi:10.1038/s41746-019-0131-z)
Supplement: Supplementary file 2 — Reporting Summary [file 41746_2019_131_MOESM2_ESM.pdf]

nature research

Corresponding author(s): Martin Stumpe and Craig Mermel

Last updated by author(s): Apr 19, 2019

Reporting Summary

Nature Research wishes to improve the reproducibility of the work that we publish. This form provides structure for consistency and transparency in reporting. For further information on Nature Research policies, see Authors & Referees and the Editorial Policy Checklist.

Statistics

For all statistical analyses, confirm that the following items are present in the figure legend, table legend, main text, or Methods section.

n/a	Confirmed	
☐	☒	The exact sample size (*n*) for each experimental group/condition, given as a discrete number and unit of measurement
☐	☒	A statement on whether measurements were taken from distinct samples or whether the same sample was measured repeatedly
☐	☒	The statistical test(s) used AND whether they are one- or two-sided<br>*Only common tests should be described solely by name; describe more complex techniques in the Methods section.*
☐	☒	A description of all covariates tested
☐	☒	A description of any assumptions or corrections, such as tests of normality and adjustment for multiple comparisons
☐	☒	A full description of the statistical parameters including central tendency (e.g. means) or other basic estimates (e.g. regression coefficient) AND variation (e.g. standard deviation) or associated estimates of uncertainty (e.g. confidence intervals)
☐	☒	For null hypothesis testing, the test statistic (e.g. *F*, *t*, *r*) with confidence intervals, effect sizes, degrees of freedom and *P* value noted<br>*Give P values as exact values whenever suitable.*
☒	☐	For Bayesian analysis, information on the choice of priors and Markov chain Monte Carlo settings
☒	☐	For hierarchical and complex designs, identification of the appropriate level for tests and full reporting of outcomes
☒	☐	Estimates of effect sizes (e.g. Cohen's *d*, Pearson's *r*), indicating how they were calculated

Our web collection on statistics for biologists contains articles on many of the points above.

Software and code

Policy information about availability of computer code

Data collection	Images used for this study were from a public source, TCGA.
Data analysis	We wrote code in Python, C++, and Angular JS to build the SMILY tool and analyze the results.

For manuscripts utilizing custom algorithms or software that are central to the research but not yet described in published literature, software must be made available to editors/reviewers. We strongly encourage code deposition in a community repository (e.g. GitHub). See the Nature Research guidelines for submitting code & software for further information.

Data

Policy information about availability of data

All manuscripts must include a data availability statement. This statement should provide the following information, where applicable:

- Accession codes, unique identifiers, or web links for publicly available datasets
- A list of figures that have associated raw data
- A description of any restrictions on data availability

Our study used images accessible from the Genomic Data Commons portal, which is based upon data generated by the TCGA Research Network.

Field-specific reporting

Please select the one below that is the best fit for your research. If you are not sure, read the appropriate sections before making your selection.

☒ Life sciences ☐ Behavioural & social sciences ☐ Ecological, evolutionary & environmental sciences

For a reference copy of the document with all sections, see nature.com/documents/nr-reporting-summary-flat.pdf

Life sciences study design

All studies must disclose on these points even when the disclosure is negative.

Sample size	Sample sizes were not calculated in advance because the large size of pathology slides resulted in hundreds of thousands of image patches for this study.
Data exclusions	Whole slide images were not specifically excluded, but slide images meeting the inclusion criteria (based on organ site) were sampled at random to create the database.
Replication	Not applicable, our experiments were "in-silico".
Randomization	Randomization was performed for the study with pathologists, based on the ratios in Table 2.
Blinding	For the study with pathologists, the pathologists were blinded to the source of the queries: random versus SMILY.

Reporting for specific materials, systems and methods

We require information from authors about some types of materials, experimental systems and methods used in many studies. Here, indicate whether each material, system or method listed is relevant to your study. If you are not sure if a list item applies to your research, read the appropriate section before selecting a response.

Materials & experimental systems

n/a	Involved in the study
☒	☐ Antibodies
☒	☐ Eukaryotic cell lines
☒	☐ Palaeontology
☒	☐ Animals and other organisms
☐	☒ Human research participants
☒	☐ Clinical data

Methods

n/a	Involved in the study
☒	☐ ChIP-seq
☒	☐ Flow cytometry
☒	☐ MRI-based neuroimaging

Human research participants

Policy information about studies involving human research participants

Population characteristics	Please refer to the publicly available The Cancer Genome Atlas.
Recruitment	Please refer to the publicly available The Cancer Genome Atlas.
Ethics oversight	TCGA tissue samples were collected with approval of local Institutional Review Boards (IRBs), with the informed consent of patients. Ethics review and IRB exemption for the use of de-identified images in this study was obtained from Quorum Review IRB (Seattle, WA).

Note that full information on the approval of the study protocol must also be provided in the manuscript.
